# Supplementary material for: New Atypical Antipsychotics in the Treatment of Schizophrenia and Depression
Source: Int J Mol Sci. 2022 Sep 13;23(18):10624. doi: 10.3390/ijms231810624 (PMC9500595; doi:10.3390/ijms231810624)

Figure S1. The three main neurotransmitter systems presumed to be involved in the development of schizophrenia. (A) DA hyperactivity in the mesolimbic DA pathway (from the ventral tegmental area (VTA) to the ventral striatum) at D_2 receptors is the cause of the positive symptoms of schizophrenia, whereas DA hypofunction in the mesocortical DA pathway is responsible for the negative symptoms. (B) Hypoactive NMDAR in the cerebral cortex leads to reduced output from GABA interneurons. This then results in disinhibition of DA neurons – the increase in DA synthesis and release in the ventral striatum. (C) The hyperactivation of 5-HT_{2A} receptors on glutamate neurons leads to the increase in glutamate release in the VTA, resulting in excess DA in the ventral striatum.

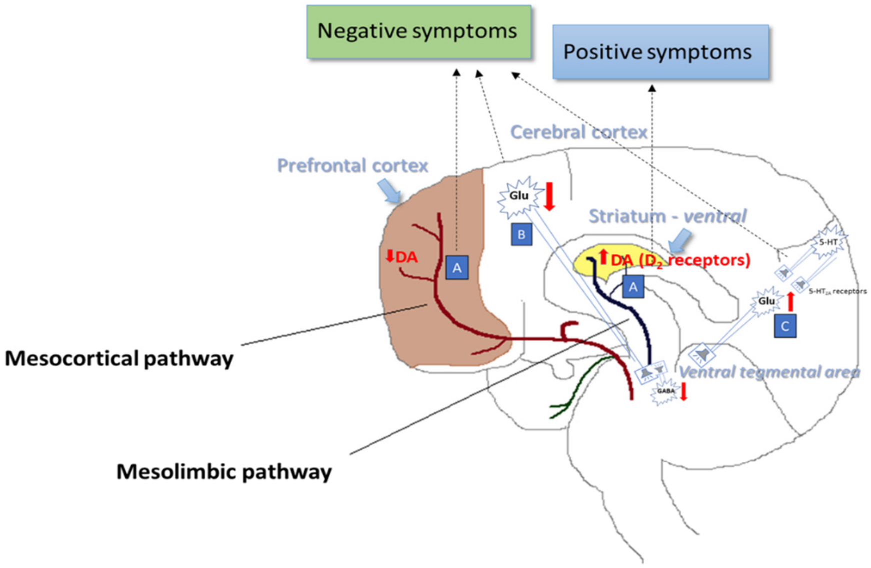

Supplement: Supplementary file 1 [file ijms-23-10624-s001.zip › ijms-1863564-supplementary/Figure S1.pdf]
