# Supplementary material for: New Atypical Antipsychotics in the Treatment of Schizophrenia and Depression
Source: Int J Mol Sci. 2022 Sep 13;23(18):10624. doi: 10.3390/ijms231810624 (PMC9500595; doi:10.3390/ijms231810624)

Figure S2. The main neurotransmitter systems presumed to be involved in the development of depression. The figure shows a simplified summary of two brain systems: the brain reward system (VTA- ventral tegmental area-nucleus accumbens (NAc) and VTA-prefrontal cortex pathways (FC)) and hippocampus (HP) - hypothalamic-pituitary-adrenal (HPA) pathway. Pro-inflammatory cytokines (TNF, IL-1, IL-6), released in response to a stress, disrupt monoaminergic and neurotrophic signalling. The VTA provides dopaminergic input to each of the limbic structures. NE and 5-HT innervate all of the regions shown.

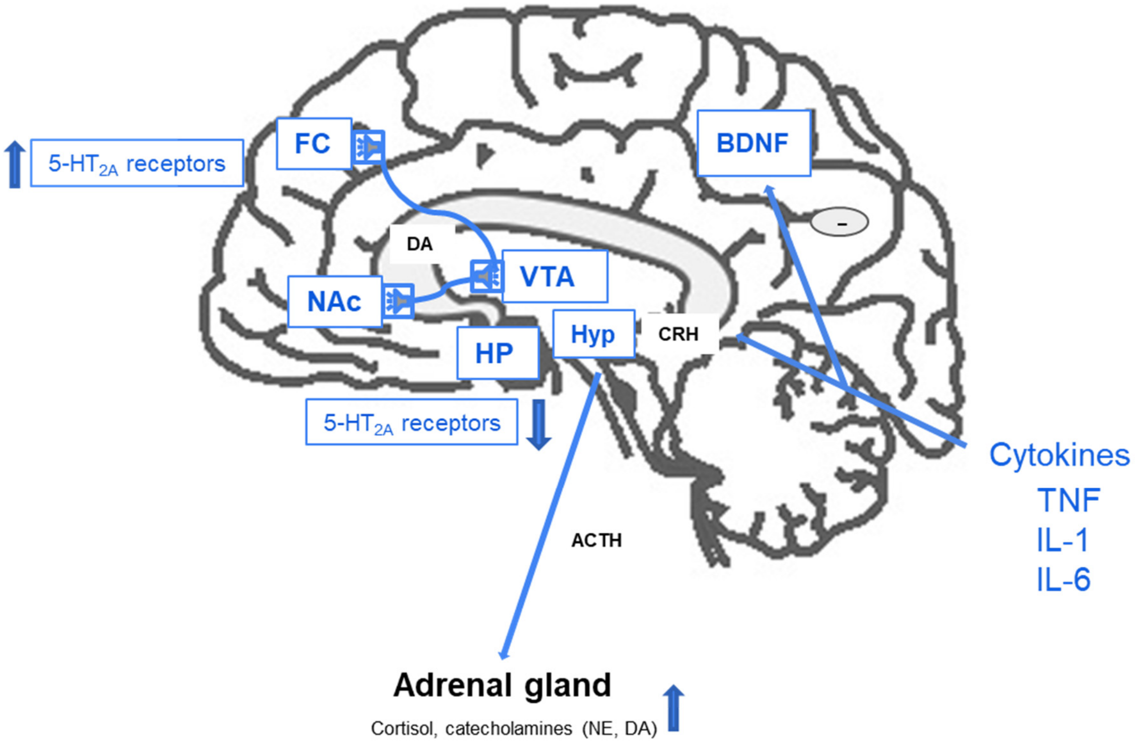

Supplement: Supplementary file 1 [file ijms-23-10624-s001.zip › ijms-1863564-supplementary/Figure S2.pdf]
